# Age Changes of Jaws and Soft Tissue Profile

**DOI:** 10.1155/2014/301501

**Published:** 2014-11-20

**Authors:** Padmaja Sharma, Ankit Arora, Ashima Valiathan

**Affiliations:** ^1^Orthodontics and Dentofacial Orthopaedics, Manubhai Patel Dental College & Hospital and Oral Research Institute, Vadodara, Gujarat 390011, India; ^2^Conservative Dentistry and Endodontics, Manubhai Patel Dental College & Hospital and Oral Research Institute, Vadodara, Gujarat 390011, India; ^3^Department of Orthodontics and Dentofacial Orthopaedics, Manipal College of Dental Sciences, Manipal, Karnataka 576104, India

## Abstract

Age-related changes of jaws and soft tissue profile are important both for orthodontists and general dentists. Mouth profile is the area which is manipulated during dental treatment. These changes should be planned in accordance with other components of facial profile to achieve ultimate aim of structural balance, functional efficacy, and esthetic harmony. Through this paper, the authors wish to discuss age changes of the hard and soft tissues of human face which would help not only the orthodontists but also oral surgeons, prosthodontists, pedodontists, and general dentists.

## 1. Introduction

Age-related changes of jaws and soft tissue profile are important both for orthodontists and general dentists. Behrents [1] reported that craniofacial growth does not stop in young adulthood but is a continuous process even into later ages. The units of change are small but change in the craniofacial skeleton has become the operational concept rather than termination of the process.

The increasing demand for adult orthodontics and orthognathic surgery increases the need to understand the facial aging process.

## 2. Growth and Profile Change: A Historical Background

The physical anthropologists in earlier days worked with dry skull. Keith and Campion [2] studied human facial growth from childhood to adulthood, using immature and mature skulls and 32 living individuals.

Hellman [3] made over 45,000 measurements of external dimensions of the face after studying 705 males and 988 females ranging from 3 to 22 years of age. He concluded that, “the infant face is transformed into that of the adult not only by increases in size, but by changes in proportion and adjustment in position as well.”

Broadbent [4] instrumented a longitudinal study of over 4000 subjects in 1929 at Case Reserve University in Ohio. The findings were presented in the form of superimposed tracings of serial cephalograms made at several stages from 1 month to adulthood. This study is known as* Bolton Brush growth study*.

Behrents [5] did an extensive adult follow-up research of subjects in the original Bolton study, analyzing 163 subjects in the age range of 17 to 83 years. He concluded that craniofacial size and shape changes continue past 17 years to the oldest ages studied. He summarized that significant sexual dimorphism existed: men are larger at all ages, they grow more, and their adult growth is more apt to persist along the same vectors of adolescent growth. On the other hand, women showed periods of increased rates of craniofacial growth, apparently related to the time of pregnancies.

## 3. Child Face

The child has a high intellectual-like forehead without coarse eyebrow ridges, with prominent cheekbones, large and wide-set eyes, and a flat face. It has a short nose, low nasal bridge, and a concave nasal profile. The face is vertically short because of small nasal part, still growing jaw bones and not yet established primary and secondary dentition. Whether a young child's head form is dolichocephalic or brachycephalic, the face itself appears more brachycephalic-like because it is still relatively wide and vertically short [6].

In a profile view, the most striking feature is lower jaw which is far retrusive than the face above. The general tendency seems to be for the mandible to grow from the more retruded to a less retruded position and this is usually true regardless of the individual facial type. The maxilla tends to be positioned in a forward direction much more slowly than does the mandible, resulting in a decrease in the convexity of the facial profile. This differential growth in an anterior direction determines the final facial type at the completion of growth [6].

## 4. Why Is There a Change in Profile?

*(i) Differential Growth: Hard Tissue/Soft Tissue.* According to Scammon's growth curve, different organs in the body grow at different times to a different amount at different rates [7]. 

*(ii) Cephalo-Caudal Gradient of Growth.* There is an axis of increased growth extending from head towards the feet. This increased gradient of growth is evident even within the face. The cranium is proportionally larger than face during birth but, postnatally, face grows more than cranium. Similarly, the mandible grows more in amount and for longer duration than maxilla [7].

*(iii) Function.* In a child, nasal part of the face is underdeveloped because of overall small body and lung size at that stage. Correspondingly, respiratory function has low demands. The nasal part of the face and the pharyngeal space has to enlarge in response to increased demands on respiratory function imparted by increasing overall body and lung size. For the nasomaxillary space to enlarge, nasomaxillary complex has to grow out from beneath the anterior cranial base. Then both the jaws have to grow to accommodate erupting deciduous and subsequently permanent dentition and enlarging muscles of mastication. These factors impart a vertical height and a depth to the face [6].

## 5. Hard Tissue Profile Changes

### 5.1. Forehead

The neurocranium grows earlier faster and to a much greater extent than facial complex. Cranial cavity completes 90% of its growth by 5 yrs of age. The young child's forehead is upright and bulbous. This region seems very large and high because the face beneath it is still relatively small. But in the following years the face enlarges much more so that the proportionate size of the forehead becomes reduced. Pneumatization of the frontal sinus is responsible for the supraorbital ridges becoming prominent and forehead becoming much more sloping [6].

### 5.2. Nasal Bone

The young child has small rounded nose that protrudes very little and is vertically quite short. The nasal bridge is quite low with the lateral bony wall of the nose being characteristically narrow and shallow. The whole nasal region of the infant is vertically shallow and the nasal floor lies close to the inferior orbital rim. The shape of the nasal bridge changes from concave to convex [6].

### 5.3. Maxilla and Mandible

Björk and Palling [8] found that during the earlier teenage years, growth of the mandible exceeds that of the maxilla resulting in straightening of the profile and retroclination of the lower incisors which may be one of the reasons for the increase in lower arch crowding at that time.

Longitudinal studies on postpubertal growth are limited. Slightly smaller jaw length increases were noted by Sarnas and Solow [9] between 21 and 26 years, Bishara et al. [10] between 25 and 46 years, and by Bondevik [11] between 22 and 33 years. Lewis et al. [12] also showed that growth in the mandible and cranial base continues into the third decade. However, Björk [13] determined the mandibular growth rate in 45 Danish males to be 3 mm between the ages of 16 and 17 years and decreased to no growth between 21 and 22 years.

Postpubertal craniofacial skeletal and dental changes were examined from lateral cephalograms of Class I males taken when subjects were 16, 18, and 20 years of age by Love et al. [14]. Mandibular growth was found to be statistically significant for the age periods of 16 to 18 years and 18 to 20 years. Growth from 16 to 18 years was greater than that from 18 to 20 years. Maxillary and mandibular growths were highly correlated at each age period. However, overall mandibular growth was approximately twice that of overall maxillary growth. Mandibular growth was found to involve an upward and forward rotation, a result of posterior vertical growth exceeding anterior vertical growth. Lower incisors were found to tip lingually with increasing age.

Foley and Mamandras [15] determined the magnitude and the direction of postpubertal mandibular and maxillary facial growth in females. The sample consisted of 37 untreated subjects who had Class I skeletal and dental characteristics and whose lateral cephalograms were taken at 14, 16, and 20 years of age. Mandibular growth was determined to be significant for the age periods of 14 to 16 years and 16 to 20 years. Overall mandibular growth was approximately twice that of the overall maxillary growth. The mandibular growth rate was found to be twice as large for age period 14 to 16 years as for age period 16 to 20 years. The increase in posterior vertical face height was slightly more than the increase in anterior vertical face height.

The mandibular plane angle decreased 1.1° during the age period of 14 to 20 years, suggesting a tendency for a closing rotation of the mandible. Mandibular incisors appeared to tip labially with advancing age. Although variable, the potential for significant maxillary and mandibular facial growth in females during late adolescence has been demonstrated.

### 5.4. Premaxilla

The anterior outline of the bony maxillary arch in the infant has a vertically convex topography. This is in contrast to the characteristic concavity this region develops in the adulthood. The alveolar bone in this area of the adult face is noticeably protrusive. Anterior contour of premaxilla is flat in infants; the differential remodeling process draws out this contour [6].

### 5.5. Age-Related Arch Width Changes

Bishara et al. [16] found that for maxillary arch, intercanine width increases between 3 and 13 yrs by 6 mm but decreases by 1.7 mm between 13 and 45 yrs. On the other hand, intermolar width increases by 2 mm between 3 and 5 yrs and by 2.2 mm between 8 and 13 yrs but decreases by 1 mm by 45 yrs of age. There is a slight decrease in arch length with age because of uprighting of the incisors.

Kanekawa and Shimizu [17] in a study on age-related changes on bone regeneration in midpalatal suture during maxillary expansion in the rat suggested difficulty of rapid palatal expansion in 52 w rats. Furthermore, age-related decrease in bone regeneration after expansion of the suture within 24 w rats may not be caused by decrease in bone matrix formation but decrease in mineralization of bone matrix. Therefore, the midpalatal suture can be expanded in the cases matured beyond pubertal growth, but more time may be necessary to regenerate mineralized bone in the suture.

In mandibular arch, intercanine width increases between 3 and 13 yrs by 3.7 mm but decreases by 1.2 mm between 13 and 45 yrs. Intermolar width increases by 1.5 mm between 3 and 5 yrs and by 1 mm between 8 and 13 yrs but decreases by 1 mm by 45 yrs of age. There is a slight decrease in arch length with age because of uprighting of the incisors and loss of leeway space by the mesial movement of the first permanent molars [16].

Mandibular intercanine width, on the average, is established by 8 years of age, that is, after the eruption of the four incisors. After the eruption of the permanent dentition, the clinician should either expect no changes or a slight decrease in arch widths [16].

A longitudinal study of arch size and form in untreated adults was performed by Harris et al. [18]. Arch lengths measured in a pure longitudinal series of untreated adults, at about 20 and again at about 55 years of age, decreased significantly with time. This was a normal, predictable function of aging. Arch widths increased, with little change across the canines, but appreciably more in the more distal regions of each arch.

### 5.6. Anterior Facial Height

The increase in anterior face height is probably largely due to continued tooth eruption. In females, the slight increase in the maxillary/mandibular plane angle may contribute to the increase in anterior face height. Sarnas and Solow [9] and Forsberg [19] suggested that the major part of the anterior face height increase in the third decade takes place in the first half of the decade. However, Bondevik [11] who reported a 1.0 mm increase in anterior face height between 22 and 33 years and Bishara et al. [10] who found a 1.9 mm increase between 25 and 46 years refute this statement. Apparently, anterior face height increase continues well into the fourth decade.

### 5.7. Posterior Facial Height

In males, the posterior facial height increases by almost as much as the anterior face height. In females, the posterior face height does not increase significantly in contrast to the anterior face height. This accounts for the slight increase in the maxillary/mandibular plane angle. However, Bishara et al. [10] found that anterior and posterior face heights increased by the same amount in females with no significant change in the mandibular plane angle from 25 to 46 years.

### 5.8. Chin

The chin is incompletely formed in the infant. The mandible of the young child is quite small and retrusive relative to the upper jaw. The anterior cranial fossa is developmentally precocious. Hence, the nasomaxillary complex is carried to a more protrusive position. The mandible, which articulates on the middle cranial fossae, is located more posteriorly. With continuing growth, the chin tends to assume forward position relative to the superior aspects of the skeletal face and the mandible grows from the more retruded to a less retruded position [6].

## 6. Components of Soft Tissue Profile

### 6.1. Nose

The soft tissue nose is short, rounded, and pug-like. The nasal bridge is low; the nasal profile is concave and the nares can be seen in a face on view. It protrudes very little and is vertically quite short [6].

The human nose continues to grow in a downward and forward direction at least until early adulthood. There does not seem to be an appreciable decrease in the rate of nasal growth which is typical for the skeletal structures. Average yearly increase of 1–1.3 mm in the overall length of the external nose is almost the same for males and females.

### 6.2. Nasal Growth and Its Contribution to Profile

In a longitudinal study, Behrents [1] concluded that the upper dorsum rotates upwards and forwards (counterclockwise) approximately 10° between 6 and 14 years of age. The lower dorsum shows both downward and backward (clockwise) and upward and forward (counterclockwise) rotation. This clearly indicated that changes in the nasal dorsum are most closely related to angulation changes of the lower dorsum, particularly during adolescence. The lower dorsum rotates downwards and backwards in persons who show greater vertical and less horizontal growth changes. Rotational changes of the lower dorsum are most closely related with vertical changes at pronasale [20].

Chaconas [21] showed that Class I subjects have more forward growth of the nasal tip than Class II subjects; Class II subjects tend to have a pronounced elevation of the dorsum and Class III subjects tend to have a concave dorsum.

Subtelny [22] first documented the downward and forward growth of the nose with maturity. The vertical dimension of the nose experiences more growth than the anteroposterior projection in both males and females. There was a spurt seen in male's nasal growth from 10 to 16 years with a peak around 13-14 years. Class II patients exhibited a more pronounced elevation of the bridge of the nose than Class I. Class I cases tended to have straighter noses. Females did not show such a spurt in growth like males but had a more steady increase in nose growth. This is of importance because an orthodontist treating a Class II girl aged 12 yrs could expect only minimal increases in nasal projection over the next few years. However, in a male of a similar age any treatment that causes upper lip retraction in combination with several mm of nose growth might produce a less than optimal final relationship between the lips and nose. When the nose is included in the profile appraisal, the soft tissue profile is seen to be increasing in convexity with progressive growth. This happens because the nose grows in a forward direction to a proportionately greater degree than the other soft tissues of the facial profile.

Wisth [23] stated that as the inclination of the nose remains constant, the profile changes must be due to increments in nose length. This growth is almost linear about 1 millimetre each year. The growth in depth is only half this amount and as it does not change the inclination of the nose, it only seems to compensate the anterior movement caused by the downward growth along the original growth axis, determined by the inclination. This growth will change the position of the tip of the nose in relation to the chin and thus change the profile convexity.

In the later stages of development, the nose usually becomes more inclined in a forward direction and the tip of the nose becomes more acute. Vertical dimension of the nose increases until 18 years of age. The upper nose height is found to increase 3 times more than the lower nose height, thereby maintaining a ratio of upper nose height to lower nose height of 3 : 1.

The skeletal facial convexity decreases in both sexes, while the soft tissue facial convexity, excluding the nose, is almost unchanged. The total facial convexity, including the nose, increases during the whole period. The result is that even if the skeletal angle indicates a straightening of the face, and the soft tissue angle shows no alterations, the profile, including the nose, shows a definite increase of the convexity. Thus, it seems that the growth of the nose is responsible for most of the profile changes [23].

On the other hand, in an individual with inherently small nose, it may be desirable to institute procedures which will cause the lips to retract. Retraction of the lips and continued facial growth may dramatically improve facial appearance.

### 6.3. Lips

#### 6.3.1. Changes in Lip Length and Thickness Associated with Growth

Both upper and lower lips grow more than the skeletal lower face in children. In both absolute and proportional terms, the lower lip grows more than the upper lip [22]. Lips grow earlier in girls than boys and in soft tissues as in the skeleton, a cephalocaudal gradient of growth is observed.

The upper lip shows rapid increase in length from age 1 to 3 yrs. The rate of growth then reduces from age 3 to 6 yrs when again an upswing occurs till the age of 15 yrs. The growth curve for the upper lip is similar to the growth curve for the general body growth curve.

Most children with lip incompetence at age 6 experience self-correction by the age of 16. Lip competence is important in terms of not only esthetics but also stability of overjet correction. In this age group 6–8, it looks as though the incompetency is due to short lips whereas it is just incomplete soft tissue growth [24].

Genecov et al. [25] showed in his study that males between the ages of 7 and 17 yrs had a greater increase in lip length than females in the same period. The males experienced little more than 2 mm in the vertical growth of the upper lip whereas in females it was less than a mm. Mamandras [26] in his study found that in females vertical lip growth was complete by 14 yrs whereas in males it leveled off at 18 yrs. Mandibular lip length increased till 16 yrs in females whereas in males it was not completed at 18 yrs.

#### 6.3.2. Lip Thickness during Growth and Maturity

In Subtelny's study [22], it was found that the upper lip attained a greater thickness in the vermillion region than over point A. This increase in thickness at the vermillion border was approximately equal to the increase in length of the lip. In both males and females, the upper lip increased in thickness from ages 1 to 14. After the age of 14 yrs, the lips continued to become thicker in males but not in females. Similarly, in the lower lip the gain in thickness was greater at vermillion border than at pogonion or point B. Lip thickness increase for males from ages 1 to 18 yrs was around 7 mm while for females it was around 6 mm.

Mamandras [26] in his study of lip thickness found that the female lip thickened till the age of 14 yrs after which it remained the same till the age of 18 yrs and beyond that it showed thinning. Males attained maximum lip thickness by age of 16 yrs; after that they too showed thinning. Nanda et al. [27] slightly differed from the above findings as he found that lip thickness increased uniformly from age 7 to 18 yrs and females attained full lip thickness by age 13 yrs with slight thinning starting then. In males, however, the thickness continued till the age of 18 yrs.

#### 6.3.3. Clinical Applications

The differential in the two sexes with respect to lip thickness implies that the treatment result of extraction therapy of the facial profile will be more noticeable in female than male patients.

Because female lips do not thicken with age, any extraction plan for females with straight to convex profiles should be cautiously considered. Lip fullness in relation to the nose which will continue to grow should also be noted [28].

In spite of progressive increase in length, both lips show a fairly constant vertical relationship to their respective alveolar processes. After the full eruption of the central incisors, there is little increase in the vertical distance between the crest of the alveolar process and the vermillion border of the lip. The lips also maintain an equally constant relationship to the incisal edges of the anterior teeth.

This is of great clinical importance because surgical overintrusion of maxilla results in an esthetically disastrous aging of the patient's face. The male profile generally was shown to straighten with age with a concomitant retrusion of the lips, whereas the female profile did not straighten nor were the lips retruded [28].

The A-P posture of the lips is also found to be closely related to their supporting hard tissue structures, that is, the teeth and alveolar processes. The maxillary-mandibular dentitions progressively become more retruded relative to its supporting skeletal bone and to the facial plane of the skeletal profile.

### 6.4. Soft Tissue Chin

Genecov et al.'s study [25] documented that soft tissue chin thickness in females from age 7 to 9 yrs was greater than males. Females only had a 1.6 mm increase upto age 18 whereas the males had a 2.4 mm increase in soft tissue drape over the chin. As a result both sexes had a similar soft tissue thickness at age 17.

In Nanda's study [29], the soft tissue thickness over the chin, symphysis thickness, and the length of the mandibular corpus, all 3 distances increased with age, the males showing the largest increments.

Till 7 years the size of the mandibular corpus was the same for both sexes and the curves progressed parallel to each other till the age of 15 when the male sample had larger increases than the female. Increased chin projection seen in the males was due to the mandibular growth than the increase in soft tissue chin thickness.

Wisth [23] showed that the change of soft tissue thickness on the chin was almost identical to that found over nasion. This meant that soft tissue changes of the chin cannot be responsible for changes in the profile convexity [23].

An old adage is that children with a large symphysis would grow up to have an even larger one. However, when there is little symphysial prominence at the chin, the soft tissue chin can make up the deficiency [30].

The soft tissue structures overlying other skeletal landmarks do not show the same pattern of change as that observed for the bony profile. The average hard tissue profile definitely tends to become straighter with age whereas the analogous soft tissue profile tends to remain comparatively stable in its convexity.

### 6.5. Soft Tissue Profile Changes from 5 to 45 Years of Age

Bishara et al. [31] in a longitudinal study concluded that the timing of the greatest changes in the soft tissue profile occurs earlier in females (10 to 15 years) than in males (15 to 25 years) and the angle of soft tissue convexity that excludes the nose expresses little change between 5 and 45 years. Of the subjects evaluated between 5 and 25 years of age, 17 demonstrated a decrease in convexity, 8 demonstrated no change, and 10 demonstrated an increase in facial convexity with growth. There was an average decrease in facial convexity between 25 and 45 years of age.

The upper and lower lips became significantly more retruded in relation to the esthetic line between 15 and 25 years of age in both males and females and similar trends continued between 25 and 45 years of age.

Torlakovic and Faerøvig [32] analysed cephalograms from same subjects in their 20 s (*T*1), 30 s (*T*2), and 40 s T(3). They concluded that during *T*2-*T*1, for males, the whole profile was displaced anteriorly and slightly superiorly and for females, the lower facial profile was displaced in a posterior and inferior direction. Greater changes occurred in the female profile than the male profile. During *T*3-*T*2, the female profile changed slightly while the male profile underwent great changes: the upper facial profile was displaced anteriorly and the lower profile was displaced posteriorly. The whole profile was displaced in the inferior direction.

Significant changes occurred in the soft tissue facial profile from the second to fourth decades. Aging of the male facial profile began 10 years later than for females; however, when the changes did occur, they were of greater magnitude. The upper facial profile was displaced in the anterior direction and the whole profile was displaced inferiorly for both sexes.

Bishara et al. [10] longitudinally evaluated untreated normal individuals (15 males and 15 females) at ages 25 and 46. The male skeletal profile tended to increase in convexity because of an increase in the prominence of the maxilla, whereas the female skeletal profile tended to increase in convexity because of a posterior rotation of the mandible.

Formby et al. [33] concluded that females showed more changes in soft and hard tissue measurements after 25 years of age than before, whereas most hard tissue changes in males had been accomplished by the age of 25 but not soft tissue changes.

### 6.6. Nasolabial Angle

With decrease in lip prominence and lowering of the nasal tip, nasolabial angle becomes more acute. As nasal tip descends and rotates, the lip descends with it in what is termed as a clockwise rotation of the nasolabial complex.

The nasolabial angle decreases slightly from 7 to 18 years in both sexes. The mean at 7 years was 107.8 ± 9.4 degrees for males and 114.7 ± 9.5 degrees for the females. At 18 years, the mean was slightly reduced to 105.8 ± 9.0 and 110.7 ± 10.9 degrees [27].

### 6.7. Mentolabial Angle

The mentolabial angle decreases slightly from 7 to 18 years in both sexes. The mean at 7 years was 125.3 ± 8.4 degrees for males and 136.1 ± 11.6 degrees for the females. At 18 years, the mean was reduced to 125.1 ± 12.9 and 127.1 ± 12.9 degrees [27].

It would be reasonable to assume that individuals would appear less protrusive as they age, due to a number of factors. The maxillary incisors are continually uprighting during adulthood and with the continued growth of the nose, repositioning of the lips, and the vertical increases, one could easily envision that the adult would appear less protrusive over time.

The mandible increases in size in both males and females, but in the male the occlusal plane tends to flatten and the gonial angle becomes more acute. The net effect is a tendency for a continued counterclockwise rotation of the mandible. In the female, more vertical change is evident and the mandible appears to be rotating clockwise [6].

The possibility that continued growth differences in males and females might suggest a greater possibility of relapse of female Class II cases than male Class II cases and of male Class III cases than female Class III cases. Conversely, male Class II and female Class III corrections might be enhanced [1].

## 7. Smile Changes with Age

As a person ages, the smile gets narrower vertically and wider transversely. The dynamic measures indicate that the muscles' ability to create a smile decreases with increasing age.

A study by Desai et al. [34] showed a decrease of 1.5 to 2 mm in maxillary incisor display during smile with increasing age. No subject in the 50 and over age group had a high smile and no subject in the 15-to-19-year group had a low smile. All dynamic measurements indicated a pattern of decreasing change from rest to smile, especially evident after ages 30 to 39 years.

van der Geld et al. [35] found that, in older subjects, maxillary lip line heights decreased significantly in all situations. Lip line heights during spontaneous smiling were reduced by approximately 2 mm. In older participants, the mandibular lip line heights also changed significantly and the teeth were displayed less during spontaneous smiling. Mandibular tooth display in the rest position increased significantly. Upper lip length increased significantly by almost 4 mm in older subjects, whereas upper lip elevation did not change significantly.

The significant increasing lip coverage of the maxillary teeth indicates that the effects of age should be included in orthodontic treatment planning.

*In Males*. The general soft tissue changes between the ages of 18–42 included the following finding: the profile straightened; the lips became more retrusive. The nose increased in size in all dimensions. There was increased soft tissue thickness at the pogonion. There was decreased upper lip thickness and slightly increased lower lip thickness [28].

*In Females.* The profile did not become straighter and the lips did not become more protrusive. The nose increased in size in all dimensions. There was decreased soft tissue thickness at the pogonion. There was upper lip thickness and slightly increased lower lip thickness [28].

Orthodontic treatment that diminishes lower facial height, reduces lip projection, decreases maxillary incisor display, or deepens the lateral nasal grooves should be avoided if possible because they hasten facial aging characteristics [28].

## 8. Features Associated with Aging

As a person ages, lower part of the face appears to lengthen, the interlabial line descends, and the number of vertical fibers in the upper lip reduces. The philtral columns become less prominent and the vermillion becomes a straight line. Jowling and increased nasolabial folds are seen. The M and W shapes of the lips may become straight and the commissures droop giving the look of a frown [28].

Crow's feet at lateral corners of the eyes, horizontal lines on the forehead, vertical corrugations overlying the glabella, vertical furrows along the upper lip, horizontal crease above the chin, and a “turkey gobbler” bag of skin sagging down the skin below the chin can also be seen.

As general loss of body weight occurs, resorption of subcutaneous adipose tissue results in surplus of skin leading to sagging, wrinkling, and creasing. The distribution of collagenous matrix changes, fibres increase in massiveness, and whole skin decreases in resilience. The fibroblasts decline in number and cellular activities. Thus, there is a decrease of hydrophilic protein mucopolysaccharides leading to shrunken facial volume. There is darkening of skin below eyes because of more visible venous plexus in the thinned suborbital hypodermis. The suborbital integument also begins to sag to form bags [6].

Orthodontic tooth movement as a result of bone modeling and remodeling also depends greatly on age-related changes of the skeleton. Cortical bone becomes denser while the spongeous bone reduces with age and the structure changes from that of a honeycomb to a network [6].

## 9. Conclusion

Child face is not a miniature form of adult face. As growth process takes place, the changes in the hard and soft tissues of the face bring about a significant change in structure and profile of the face.

## Clinical Relevance

Knowledge about the age changes of jaws and soft tissue profile will help the dentists to aid in decision making to arrive at a comprehensive treatment plan and achieve better treatment efficiency.
